# Humoral Responses Elicited after a Fifth Dose of SARS-CoV-2 mRNA Bivalent Vaccine

**DOI:** 10.3390/v15091926

**Published:** 2023-09-15

**Authors:** Alexandra Tauzin, Guillaume Beaudoin-Bussières, Mehdi Benlarbi, Manon Nayrac, Yuxia Bo, Gabrielle Gendron-Lepage, Halima Medjahed, Josée Perreault, Laurie Gokool, Pascale Arlotto, Chantal Morrisseau, Cécile Tremblay, Daniel E. Kaufmann, Valérie Martel-Laferrière, Inès Levade, Marceline Côté, Renée Bazin, Andrés Finzi

**Affiliations:** 1 Centre de Recherche du CHUM, Montreal, QC H2X 0A9, Canada; 2 Département de Microbiologie, Infectiologie et Immunologie, Université de Montréal, Montreal, QC H2X 0A9, Canada; 3 Department of Biochemistry, Microbiology and Immunology, and Centre for Infection, Immunity and Inflammation, University of Ottawa, Ottawa, ON K1H 8M5, Canada; 4 Héma-Québec, Affaires Médicales et Innovation, Quebec, QC G1V 5C3, Canada; 5 Département de Médecine, Université de Montréal, Montreal, QC H3T 1J4, Canada; 6 Division of Infectious Diseases, Department of Medicine, Lausanne University Hospital and University of Lausanne, 1011 Lausanne, Switzerland; 7 Laboratoire de Santé Publique du Québec, Institut National de Santé Publique du Québec, Sainte-Anne-de-Bellevue, QC H9X 3R5, Canada

**Keywords:** coronavirus, COVID-19, SARS-CoV-2, spike glycoproteins, omicron variants, hybrid immunity, ADCC, neutralization

## Abstract

While an important part of the world’s population is vaccinated against SARS-CoV-2, new variants continue to emerge. We observe that even after a fifth dose of the mRNA bivalent vaccine, most vaccinated individuals have antibodies that poorly neutralize several Omicron subvariants, including BQ.1.1, XBB, XBB.1.5, FD.1.1, and CH.1.1. However, Fc-effector functions remain strong and stable over time against new variants, which may partially explain why vaccines continue to be effective. We also observe that donors who have been recently infected have stronger antibody functional activities, including neutralization and Fc-effector functions, supporting the observations that hybrid immunity leads to better humoral responses.

## 1. Introduction

Rapidly after the emergence of the severe acute respiratory syndrome coronavirus 2 (SARS-CoV-2), two mRNA vaccine platforms, based on the ancestral Spike (Wuhan strain), were developed and used to vaccinate an important proportion of the world’s population [1,2,3]. However, since the beginning of the pandemic, new variants have frequently appeared, challenging vaccine efficacy. In November 2021, the SARS-CoV-2 Omicron BA.1 variant was first reported [4]. Due to the large number of mutations in its Spike glycoprotein (Table 1), conferring a higher transmission rate and stronger resistance to vaccination, it rapidly became the dominant circulating strain in the world [5,6,7]. At that time, the administration of a third dose of mRNA vaccine greatly increased humoral responses against BA.1 [8,9]. However, several Omicron subvariants, more resistant to humoral responses, have successively emerged, such as BA.2, BA.4, and BA.5 [10,11,12,13]. These variants were replaced by their descendants. From the BA.2 variant arose XBB, then XBB.1.5, which then mutated into FD.1.1. CH.1.1 also descends from BA.2, but from a different lineage, and BQ.1.1 descends from BA.5 [14]. Several recent studies have shown that these variants are increasingly resistant to neutralization because of the numerous mutations accumulated in the Spike glycoproteins (see Table 1) [10,15,16,17]. 

Since many of these variants are in circulation worldwide, health authorities recommended additional boosts, notably for populations at risk, to maintain the population’s immunity [18,19]. In addition, to increase the efficacy of vaccines against the new variants, bivalent vaccines, expressing both the ancestral and an Omicron variant Spike (BA.1 or BA.4/5), have been developed by Pfizer and Moderna. However, several studies have shown that these new bivalent vaccines only weakly increase humoral responses against the new subvariants compared to the monovalent vaccine, and poor neutralizing responses against Omicron variants were still observed after vaccination with the bivalent mRNA vaccine [17,20,21,22]. Also, due to the high transmissibility of the Omicron subvariants and low humoral responses, the vaccine provides weak protection against infection [23]. Thus, many studies have shown that breakthrough infections (BTIs, i.e., infection of a vaccinated individual) frequently occur and a significant part of the population has been infected at least once [24,25,26]. However, this increase in the number of BTIs is not accompanied by a significant increase in hospitalizations or deaths, indicating that immune memory elicited by previous infections and/or vaccination are still effective in preventing severe forms of COVID-19 despite weak neutralizing responses [27,28,29]. 

In this study, we measured humoral responses against different SARS-CoV-2 variants, including several circulating Omicron subvariants, elicited three/four weeks and four months after a fourth dose and three/four weeks after a fifth dose of mRNA vaccine. The study was conducted in a cohort of donors who mainly received a monovalent vaccine as the fourth dose and a bivalent mRNA vaccine as the fifth dose. We measured several parameters of the humoral responses, including the level of IgG induced and their avidity. We also measured their capacity to recognize several Omicron Spikes. Finally, we assessed the capacity of these antibodies to neutralize and mediate antibody-dependent cellular cytotoxicity (ADCC) activity against several Omicron subvariants. We also measured the impact of a recent BTI on humoral responses.

## 2. Materials and Methods

### 2.1. Ethics Statement

The study was conducted in accordance with the Declaration of Helsinki in terms of informed consent and approval by an appropriate board. The protocol was approved by the Ethics Committee of the CHUM (19.381, approved on 28 February 2022).

### 2.2. Human Subjects

**Figure 1:**
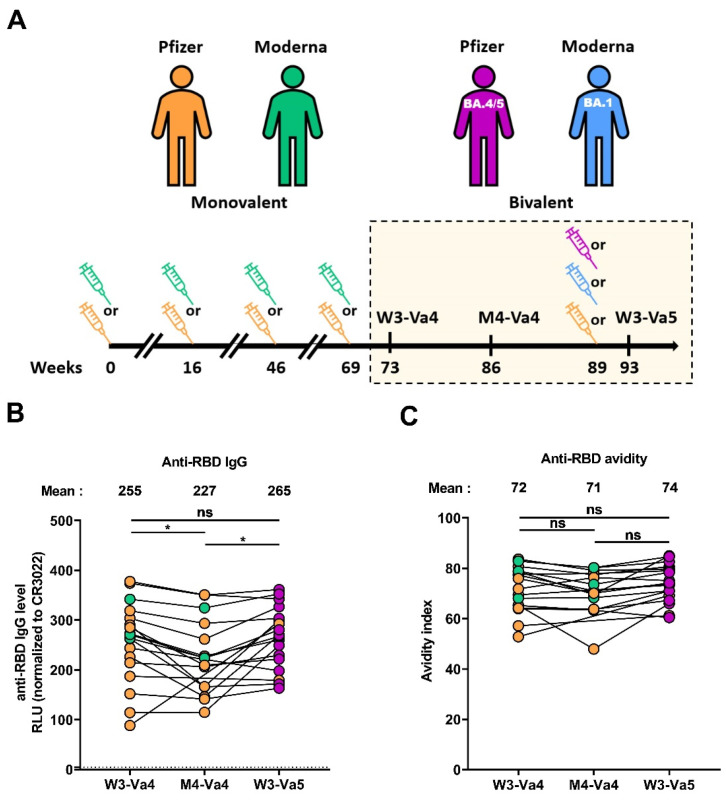
**RBD-specific IgG and relative avidity elicited after the fourth and fifth doses of mRNA vaccine.** (**A**) SARS-CoV-2 vaccine cohort design. The yellow box represents the period under study. (**B**,**C**) Indirect ELISAs were performed by incubating plasma samples with recombinant SARS-CoV-2 RBD protein. Anti-RBD IgG binding was detected using HRP-conjugated antihuman IgG. (**B**) RLU values obtained were normalized to the signal obtained with the anti-RBD CR3022 mAb present in each plate. (**C**) The avidity index corresponded to the value obtained with the stringent (8 M urea) ELISA divided by that obtained without urea. Individuals vaccinated with a Pfizer monovalent, Moderna monovalent, Pfizer bivalent (BA.4/5), or Moderna bivalent (BA.1) dose are represented by orange, green, purple, and blue points, respectively. Seropositivity thresholds are plotted. (* *p* < 0.05; ns, nonsignificant.)

The study was conducted in 18 individuals, 11 females and 7 males (age range: 51–64 years). Blood samples were collected 29 days (W3-Va4) and 124 days (M4-Va4) after the fourth dose, and 22 days (W3-Va5) after the fifth dose of mRNA vaccine. We do not include other specific criteria, such as number of patients (sample size), sex, clinical, or demographic data, for admission to the cohort. Characteristics of the cohort are summarized in Table 2 and Figure 1A.

### 2.3. Plasma Samples and Antibodies

Plasmas were isolated by Ficoll density gradient from blood samples. Plasmas were heat-inactivated for 1 h at 56 °C and stored at −80 °C until use. We used plasma samples from uninfected/unvaccinated donors collected before the pandemic as negative controls in ELISA, ADCC, and cytometry assays, and to calculate the seropositivity threshold. The CR3022 monoclonal antibody (mAb) (a receptor-binding domain (RBD)-specific monoclonal antibody) and the CV3-25 mAb (a conformationally independent S2-specific mAb) were used as positive controls in ELISA using the ancestral RBD and flow cytometry assays, respectively [10,30,31,32]. We used as secondary antibodies (Abs) a Horseradish peroxidase (HRP)-conjugated Abs that detect the Fc region of human IgG (Invitrogen) in ELISA assays and Alexa Fluor-647-conjugated goat antihuman antibodies detecting all Ig isotypes (antihuman IgM, IgG, IgA; Jackson ImmunoResearch Laboratories, cat # 109-605-064) in flow cytometry experiments.

### 2.4. Plasmids

The plasmids encoding the SARS-CoV-2 D614G, BA.1, BA.4/5, and BQ.1.1 Spike variants were previously described [10,20,33]. The pNL4.3 R-E-Luc plasmid was obtained from the NIH AIDS Reagent Program (cat# 3418). The pIRES2-EGFP-expressing plasmid was purchased from Clontech, Mountain View, CA, USA (cat# 6029-1). The plasmids encoding XBB, XBB.1.5, CH.1.1, and FD.1.1 Spikes were generated by overlapping PCR. Sequences were verified by Sanger sequencing. 

### 2.5. Protein Expression and Purification

To produce SARS-CoV-2 Spike RBD WT proteins, we transfected FreeStyle 293 F cells (Invitrogen) with the plasmid coding for SARS-CoV-2 Spike RBD WT using an ExpiFectamine 293 transfection reagent, as directed by the manufacturer (Invitrogen). One week later, cells were pelleted and discarded. Supernatants were collected and then filtered using a 0.22 μm filter (Thermo Fisher Scientific, Waltham, MA, USA). The recombinant RBD proteins were purified using nickel-affinity columns, as directed by the manufacturer (Invitrogen). The RBD preparations were dialyzed against phosphate-buffered saline (PBS). We assessed the purity of the recombinant proteins by loading on SDS-PAGE gels and staining with Coomassie Blue. The RBD proteins were stored in aliquots at −80 °C until further use. 

### 2.6. Cell Lines

The 293T human embryonic kidney (obtained from ATCC) and 293T-ACE2 cell lines were maintained in Dulbecco’s modified Eagle’s medium (DMEM) (Wisent) supplemented with 5% fetal bovine serum (FBS) (VWR) and 100 μg/mL of penicillin–streptomycin (Wisent). CEM.NKr CCR5 + cells (NIH AIDS reagent program) and CEM.NKr CCR5 + cells stably expressing the SARS-CoV-2 Spike glycoproteins (D614G, BA.1 or BA.4/5) were maintained in Roswell Park Memorial Institute (RPMI) 1640 medium (GIBCO) containing 10% FBS and 100 μg/mL of penicillin–streptomycin. The 293T-ACE2 and CEM.NKr CCR5+ cells stably expressing the SARS-CoV-2 Spike glycoproteins were previously reported [31,34,35]. The 293T, 293T-ACE2, and CEM.NKr CCR5 + cell lines were maintained at 37 °C under 5% CO_2_. FreeStyle 293 F cells were grown in FreeStyle 293F medium (Invitrogen) to a density of 10^6^ cells/mL at 37 °C with 8% CO_2_ under regular agitation (135 rpm).

### 2.7. Anti-Nucleocapsid (N) Assay

We used a previously described ELISA to measure the level of anti-N antibodies [36]. Briefly, recombinant Nucleocapsid (Centre National en Électrochimie et en Technologies Environnementales Inc., Shawinigan, QC, Canada) was coated in 96-well microplates (50 μL/well) at a concentration of 0.25 μg/mL. The plasma samples were incubated for 1 h at room temperature (RT) at a 1/100 dilution. The plates were then washed, and antihuman polyvalent IgA + IgG + IgM (H + L)-HRP conjugates were added as a secondary antibody. The plates were incubated for 1 h at RT, washed, and 100 µL of 3,3′,5,5′-Tetramethylbenzidine (TMB, ESBE Scientific) were added to the plates. The plates were incubated for 20 min at RT and then 100 µL of H_2_SO_4_ 1N (Fisher Scientific) were added to stop the colorimetric reaction. The plates were read within 30 min at 450 nm using a Synergy H1 microplate reader (Bio-Tek).

### 2.8. Anti-RBD ELISA and Avidity Assays

We prepared the SARS-CoV-2 Spike ancestral RBD proteins at a concentration of 2.5 μg/mL in PBS and then adsorbed 50 μL/well to plates (MaxiSorp Nunc, Thermo Fisher Scientific, Waltham, MA, USA) overnight at 4 °C. The next day, the supernatants were removed and blocking buffer (Tris-buffered saline (TBS) containing 0.1% Tween20 and 2% BSA) were added to the coated wells for 1 h at RT. We next washed the wells with a washing buffer (TBS containing 0.1% Tween20) four times. After washing, we incubated the wells with the CR3022 mAb (50 ng/mL) or a 1/250 dilution of plasma diluted in a solution of blocking buffer (0.1% BSA) for 90 min at RT. Of note, this dilution was selected based on previous studies that showed that this single dilution was sufficient to accurately measure binding and avidity because it allows the measurement of a wide range of antibody levels and results in a signal that is not saturating [31,37]. The plates were washed with a washing buffer 4 times. After washing, we incubated the plates for 1 h at RT with secondary Abs prepared in a diluted solution of blocking buffer (0.4% BSA). The plates were next washed 4 times. For the avidity assay, half of the plates were washed at all steps with a chaotropic agent (8M of urea) added to the washing buffer, and not the other half [37]. HRP enzyme activity was determined after the addition of a 1:1 mix of Western Lightning oxidizing and luminol reagents (Perkin Elmer Life Sciences, Waltham, MA, USA). We used a LB942 TriStar luminometer (Berthold Technologies, Bad Wildbad, Germany) to measure light emission. We used the signal obtained with the CR3022 mAb in absence of urea, and we tested in each plate for normalization. The seropositivity threshold was established using the following formula: mean of prepandemic SARS-CoV-2-negative plasma + (3 standard deviations of the mean of prepandemic SARS-CoV-2-negative plasma).

### 2.9. Cell-Surface Staining and Flow Cytometry Analysis

To assess the binding capacity of the plasma, we transfected 293T cells with a plasmid encoding the full-length SARS-CoV-2 Spikes. To distinguish transfected cells, we cotransfected the Spike expressor with a plasmid encoding the GFP (pIRES2-eGFP). Transfections were done using the calcium–phosphate method. The next day, we changed the media of the transfected cells. Two days post-transfection, cells were collected and stained for 45 min at 37 °C with the CV3-25 mAb (5 μg/mL) as control, or with plasma diluted in PBS (1/250 dilution). The cells were then washed with PBS and stained with secondary Ab (AlexaFluor-647-conjugated goat antihuman IgM, IgG, IgA diluted in PBS (1/800 dilution)). The percentage of Spike-expressing cells (GFP + cells) was determined by gating the living cell population based on the viability dye staining (Aqua Vivid, Invitrogen, Waltham, MA, USA). We used the LSRFortessa cytometer (BD Biosciences, Franklin Lakes, NJ, USA) to acquire data. We performed data analysis using FlowJo v10.8.0 (Tree Star). The CV3-25 mAb, that efficiently recognizes all SARS-CoV-2 Spike variants, was used to normalize Spike expression. The median fluorescence intensities (MFIs) obtained with plasma were normalized to the MFIs obtained with CV3-25 and presented as percentage of CV3-25 binding. The seropositivity threshold was established using the following formula: mean of prepandemic SARS-CoV-2-negative plasma + (3 standard deviations of the mean of prepandemic SARS-CoV-2-negative plasma).

### 2.10. Antibody-Dependent Cellular Cytotoxicity Assay

To evaluate ADCC activity against the SARS-CoV-2 Spike, we used parental CEM.NKr CCR5 + cells mixed with CEM.NKr CCR5 + cells expressing the SARS-CoV-2 D614G, BA.1, or BA.4/5 Spike glycoproteins at a 1:1 ratio [38]. The CEM.NKr cells, used as target cells, were stained for viability (AquaVivid) and with a cellular dye (cell-proliferation dye eFluor670; Thermo Fisher Scientific) to discriminate them during acquisition. PBMCs were used as effector cells and were stained with another cellular marker (cell-proliferation dye eFluor450; Thermo Fisher Scientific). Plasmas diluted 1/500 were added to the target cells. Of note, this dilution was selected based on a previous study which optimized this assay [35]. Target (with plasma) and effectors cells were then mixed at a 1:10 ratio and incubated for 5 h at 37 °C and 5% CO_2_. After incubation, cells were fixed with PFA 2%. Since CEM.NKr-expressing Spikes also express GFP, ADCC activity was calculated using the formula: [(% of GFP + cells in Targets plus Effectors) − (% of GFP + cells in Targets plus Effectors plus plasma]/(% of GFP + cells in Targets) × 100 by gating on transduced live target cells. All samples were acquired on an LSRII cytometer (BD Biosciences) and data analysis was performed using FlowJo v10.5.3 (Tree Star). The seropositivity threshold was established using the following formula: mean of prepandemic SARS-CoV-2-negative plasma + (3 standard deviations of the mean of prepandemic SARS-CoV-2-negative plasma).

### 2.11. Virus Neutralization Assay

To produce SARS-CoV-2 pseudoviruses, 293T cells were cotransfected with the lentiviral vector pNL4.3 R-E− Luc and a plasmid encoding the indicated full-length Spike glycoprotein (ratio 10/1) using the calcium–phosphate method. Two days after the transfection, the supernatants were collected and harvested to remove cell debris, and stored at −80 °C until use in subsequent experiments. For the neutralization assay, 293T-ACE2 were used as target cells. The day before infection, 1 × 10^4^ cells/well were seeded in 96-well luminometer-compatible tissue-culture plates (PerkinElmer, Waltham, MA, USA). The next day, several plasma dilutions (1/50; 1/250; 1/1250; 1/6250; 1/31,250) were incubated with the different SARS-CoV-2 pseudoviruses for 1h at 37 °C. After incubation, the mixtures were added to the 293T-ACE2 target cells. Cells were then incubated for 48 h at 37 °C. Two days postinfection, supernatants were removed and 30 μL of passive lysis buffer were added to the cells (Promega, Madison, WI, USA) followed by one freeze–thaw cycle to lyse the cells. Luciferase activity of each well was measured after the addition of 100 μL of luciferin buffer (15 mM MgSO_4_, 15 mM KH_2_PO_4_ (pH 7.8), 1 mM ATP, and 1 mM dithiothreitol) and 50 μL of 1 mM d-luciferin potassium salt (Prolume). We used a LB942 TriStar luminometer (Berthold Technologies, Bad Wildbad, Germany) to acquire the data. The neutralization half-maximal inhibitory dilution (ID_50_) represents the plasma dilution to inhibit 50% of the infection of 293T-ACE2 cells by pseudoviruses.

### 2.12. Statistical Analysis

We used GraphPad Prism version 9.5.1 (GraphPad, San Diego, CA, USA) to analyze our data. In all the figures, every point represents a biologically independent sample. We tested every dataset for statistical normality and subsequently applied the appropriate (parametric or nonparametric) statistical test. The *p* values < 0.05 were considered significant; significance values are indicated as * *p* < 0.05, ** *p* < 0.01, *** *p* < 0.001, **** *p* < 0.0001, and n.s., nonsignificant.

## 3. Results

### 3.1. Characteristics of the Cohort

To monitor humoral responses elicited after the fourth and fifth doses of the SARS-CoV-2 mRNA vaccine, we collected plasma samples from 18 donors (11 females and 7 males). The median age of the donors was 59 years (interquartile range: 51–64 years). Plasma samples were collected at three different time points, three/four weeks after the fourth dose (W3-Va4), four months after the fourth dose (M4-Va4), and three/four weeks after the fifth dose of mRNA vaccine (W3-Va5). All the donors were vaccinated with a fourth dose of monovalent SARS-CoV-2 mRNA vaccine (from Pfizer or Moderna), and mainly with a fifth dose of bivalent mRNA vaccine. Characteristics of the cohort are summarized in Table 2 and Figure 1A. 

### 3.2. RBD-Specific IgG and Associated Avidity

We first measured the level of anti-RBD immunoglobulin G (IgG) using a previously described ELISA [31,34,39]. Three weeks after the fourth dose (W3-Va4), we measured a high level of IgG recognizing the RBD that was slightly lower four months after the fourth dose (Figure 1B). The fifth dose of vaccine led to an increase in the level of Abs, similar to that achieved after the fourth dose. We also measured the avidity of these IgGs, an important parameter of the humoral responses because it reflects antibody-affinity maturation occurring in the germinal centers [37] and could be associated to better functions of the antibodies, such as neutralization and Fc-effector functions, by improving interaction with its cognate epitope [40,41]. For this, we used a previously described ELISA assay with washing steps having, or not having, a chaotropic agent (urea 8 M) [37,42]. Three weeks after the fourth dose, donors had antibodies with a high level of avidity, since up to 70% of the antibodies remained detectable after washing with 8 M urea (Figure 1C). The avidity remained stable four months after the fourth dose and was not further increased by the fifth dose, suggesting that antibodies reached their highest avidity level after the fourth dose, and this remained stable over time.

### 3.3. Recognition of SARS-CoV-2 Spike Variants by Plasma from Vaccinated Individuals

**Figure 2:**
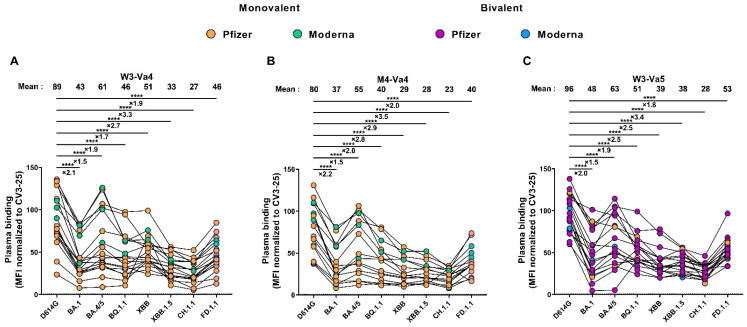
**Recognition of different SARS-CoV-2 Spikes.** (**A**–**C**) The 293T cells were transfected with the indicated full-length S, stained with the CV3-25 mAb or with plasma from vaccinated individuals collected at W3-Va4 (**A**), M4-Va4 (**B**), and W3-Va5 (**C**), and analyzed by flow cytometry. The values represent the MFI normalized by CV3-25 mAb binding. Individuals vaccinated with a Pfizer monovalent, Moderna monovalent, Pfizer bivalent (BA.4/5), or Moderna bivalent (BA.1) dose are represented by orange, green, purple, and blue points, respectively. Seropositivity thresholds are plotted. (**** *p* < 0.0001).

We also measured the capacity of plasma to recognize different SARS-CoV-2 Spikes after the fourth and fifth doses of vaccine. Three weeks after the fourth dose, the D614G Spike was efficiently recognized by all plasma (Figure 2A). We observed that all the Omicron subvariant Spikes were less-efficiently recognized than the D614G Spike, with the BA.4/5 Spike as the best Omicron variant recognized, and the CH.1.1 Spike as the least-recognized one (3.3 times less well-recognized compared with D614G). Four months after the fourth dose, the level of recognition of all the tested Spikes slightly decreased (Figure 2B). The fifth dose of mRNA vaccine boosted the levels of Spike recognition to those observed after the fourth dose (Figure 2C). At M4-Va4 and W3-Va5, the difference in the level of recognition between the Omicron subvariants and the D614G Spike was similar to that observed at W3-Va4, from 1.5 to 3.5 times less well-recognized.

### 3.4. Neutralizing Activity against Different SARS-CoV-2 Spikes

**Figure 3:**
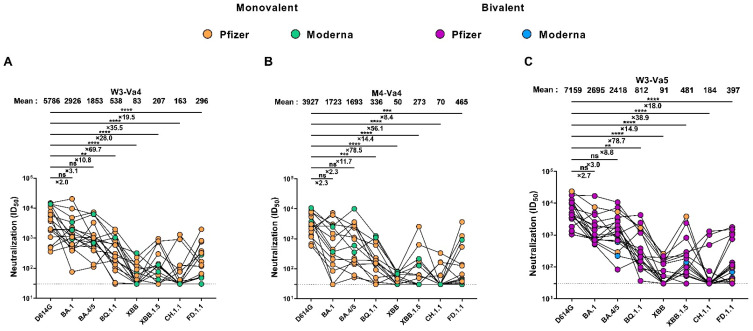
**Neutralization of different SARS-CoV-2 Spikes.** (**A**–**C**) Neutralization activity was measured by incubating pseudoviruses bearing SARS-CoV-2 Spike glycoproteins, with serial dilutions of plasma from vaccinated individuals collected at W3-Va4 (**A**), M4-Va4 (**B**), and W3-Va5 (**C**) for 1 h at 37 °C before infecting 293T-ACE2 cells. Neutralization half-maximal inhibitory serum dilution (ID_50_) values were determined using a normalized nonlinear regression using GraphPad Prism software. Individuals vaccinated with a Pfizer monovalent, Moderna monovalent, Pfizer bivalent (BA.4/5), or Moderna bivalent (BA.1) dose are represented by orange, green, purple, and blue points, respectively. Limits of detection are plotted. (** *p* < 0.01; *** *p* < 0.001; **** *p* < 0.0001; ns, nonsignificant.)

We evaluated the neutralization activity of the plasma against pseudoviral particles bearing Spikes from SARS-CoV-2 variants. As expected, all plasma strongly neutralized the D614G Spike three weeks after the fourth dose of mRNA vaccine (Figure 3A). In agreement with Spike recognition by plasma (Figure 2 and Figure S1), all the Omicron subvariants were less neutralized than the D614G Spike, with the BA.1 Spike the most efficiently neutralized, and the XBB Spike the most resistant to neutralization by plasma from vaccinated individuals. We observed a strong correlation between the capacity of the plasma to recognize and neutralize the Spikes (Figure 3A and Figure S1A). While the differences in Spike recognition between D614G and Omicron variants ranged from 1.5 to 3.5 times, the differences in the capacity to neutralize the different Spikes were more pronounced. Indeed, the BA.1 Spike was two times less-efficiently neutralized than the D614G Spike, whereas the XBB Spike was 70 times more resistant to neutralization than the D614G Spike. At M4-Va4, we observed a decrease in the level of neutralizing activity, with the exception of XBB.1.5 and FD.1.1, for which there was a slight increase (Figure 3B). The fifth dose boosted the level of neutralization for all tested Spikes (Figure 3C). However, major differences in the neutralizing capacity between D614G and the different Omicron Spikes were still observed, similar to that measured after the fourth dose of the SARS-CoV-2 mRNA vaccine (Figure 3A,C and Figure S1A,C). 

### 3.5. Fc-Effector Functions Elicited after the Fourth and the Fifth Doses of mRNA Vaccine

**Figure 4:**
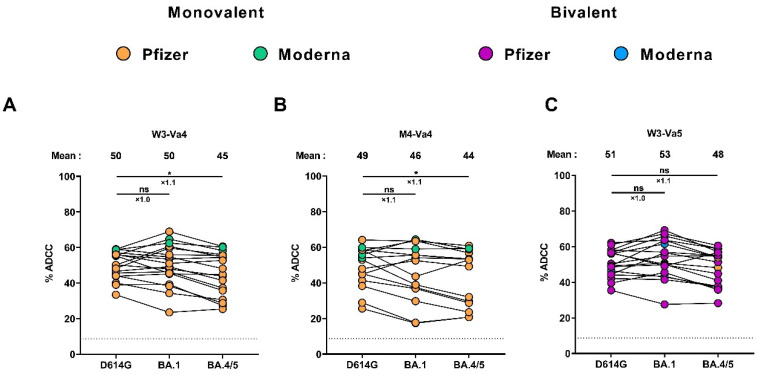
**ADCC activity against different SARS-CoV-2 Spikes.** (**A**–**C**) ADCC activity against the indicated full-length Spike was measured by incubating CEM.NKr parental cells and CEM.NKr-Spike cells (target cells) with PBMCs (effector cells) and with plasma from vaccinated individuals collected at W3-Va4 (**A**), M4-Va4 (**B**), and W3-Va5 (**C**) before being analyzed by flow cytometry. Individuals vaccinated with a Pfizer monovalent, Moderna monovalent, Pfizer bivalent (BA.4/5), or Moderna bivalent (BA.1) dose are represented by orange, green, purple, and blue points, respectively. Seropositivity thresholds are plotted. (* *p* < 0.05; ns, nonsignificant.)

To evaluate Fc-effector functions elicited after SARS-CoV-2 mRNA vaccination, we used an established ADCC assay using CEM. NKr cells stably expressing the D614G, Omicron BA.1, or BA.4/5 Spikes [34,35,38,39,43]. At W3-Va4, the same level of ADCC activity was observed against cells expressing the D614G and BA.1 Spikes (Figure 4A). Slightly less ADCC was measured against cells expressing the BA.4/5 Spike. However, the difference in ADCC activity between D614G and BA.4/5 was less pronounced than that observed for Spike recognition and neutralization (Figure 2 and Figure 3). We observed a small decrease in ADCC activity four months after the fourth dose compared to three weeks after the fourth dose, and the fifth dose reached an ADCC level to a similar level as after the fourth dose (Figure 4A–C). As observed, at W3-Va4, the differences in ADCC activity between D614G and BA.1 or BA.4/5 Spikes were less important than that observed for Spike recognition and neutralization at M4-Va4 and W3-Va5. At the three time points, strong correlations between Spike recognition by plasma and ADCC activity were observed (Figure S2).

### 3.6. Recent Infection Leads to Antibodies with Better Functional Activities

**Figure 5:**
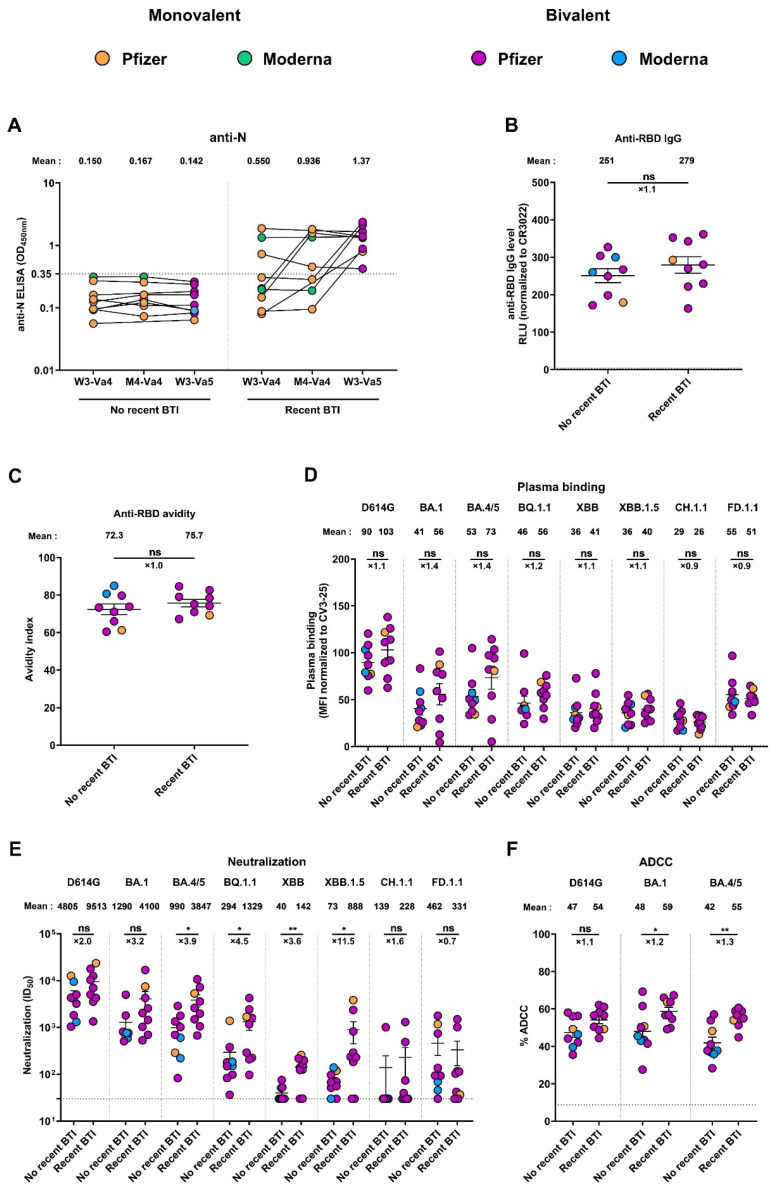
**Effect of recent BTI on humoral responses elicited after the fifth dose of mRNA vaccine.** (**A**) Anti-N level was measured after the fourth and fifth doses of SARS-CoV-2 vaccine in plasma from vaccinated donors by ELISA. (**B**–**F**) Humoral responses were measured on plasma collected three weeks after the fifth dose (W3-Va5). (**B**,**C**) Anti-RBD IgG binding was measured by indirect ELISA with recombinant SARS-CoV-2 RBD protein. (**B**) RLU values obtained were normalized to the signal obtained with the anti-RBD CR3022 mAb present in each plate. (**C**) The avidity index corresponded to the value obtained with the stringent (8 M urea) ELISA divided by that obtained without urea. (**D**) Full-length Spike recognition was measured by staining of 293T cells transfected with the indicated Spike with plasma and analyzed by flow cytometry. The values represent the MFI normalized by CV3-25 Ab binding. (**E**) Neutralizing activity was measured by incubating pseudoviruses bearing SARS-CoV-2 Spike glycoproteins with serial dilutions of plasma for 1 h at 37 °C before infecting 293T-ACE2 cells. (**F**) ADCC activity against the indicated full-length Spike with plasma was measured by flow cytometry. Individuals vaccinated with a Pfizer monovalent, Pfizer bivalent (BA.4/5), or Moderna bivalent (BA.1) dose are represented by orange, purple, and blue points, respectively. Seropositivity thresholds and limits of detection are plotted. Error bars indicate means ± SEM (* *p* < 0.05; ** *p* < 0.01; ns, nonsignificant).

Breakthrough infection is defined as the infection of a vaccinated individual [44,45]. To evaluate the impact of recent BTI on the humoral responses induced after the fifth dose, we measured the level of anti-nucleocapsid (N) Abs at W3-Va4, M4-Va4, and W3-Va5, and divided the cohort in two groups, depending on if they had a recent BTI or not (Figure 5A). Donors were considered to have a recent BTI when their anti-N level was higher than the cut-off (0.35) at least at one time point and/or when a significant increase in the anti-N Abs level between W3-Va4 and M4-Va4 or between M4-Va4 and W3-Va5 was observed (a ratio of M4-Va4/W3-Va4 and/or ratio of W3-Va5/M4-Va4 higher than 1.5), according to a described analytical approach based on the ratio of anti-N absorbance [36]. We observed that 9 donors of the 18 had an anti-N level higher than the cut-off (0.35) at W3-Va5, indicating they had a recent BTI, while for the other 9 donors, the anti-N level remained below the cut-off at all time points. (Figure 5A). Characteristics of the two groups are summarized in Table 3.

When comparing humoral responses between the two groups, we did not observe significant differences in the level of anti-RBD IgG between donors who had recent BTIs or not (Figure 5B). We also noted that the avidity of these IgGs for the RBD was similar in both groups (Figure 5C). Similarly, the different SARS-CoV-2 Spikes were comparably recognized in both groups after the fifth dose, with differences in recognition between donors with or without recent BTIs ranging from 0.9 to 1.4 times for all Spikes (Figure 5D). In contrast, the functional activities of the antibodies against the variants were increased in donors with recent BTIs. Indeed, we observed that, for the D614G Spike, both neutralization and ADCC activities were higher (2.0 and 1.1 times, respectively) in donors with recent BTIs, although these differences were not significant (Figure 5E,F). These differences were more pronounced for the Omicron Spike variants, where we observed that donors with recent BTIs had significantly higher neutralization and ADCC activities than donors without BTIs (with the exception of BA.1, CH.1.1, and FD.1.1 for neutralization, which were not significant), suggesting that these donors were infected by these circulating variants. We also noted that the differences among Spike variants were more pronounced for neutralization than for ADCC activity.

## 4. Discussion

Since the beginning of the pandemic, many variants have emerged and succeeded each other. Currently, different Omicron subvariants, including XBB.1.5, XBB, FD.1.1, and CH.1.1, are circulating and will most likely be replaced by new variants within weeks or months. Most studies, including this one, showed that the new variants have different degrees of resistance to neutralization induced by vaccination. However, although there is an important circulation of the variants in the population, no increase in the number of severe forms of COVID-19 were observed, indicating that the vaccines remain effective [16,20,29,46,47]. 

In our study, we observed poor neutralizing activity against several Omicron Spikes after a fourth and a fifth dose of the mRNA vaccine, and that the differences in neutralizing capacity between different Spikes were pronounced. In contrast, the ADCC activity measured against two Omicron Spikes (BA.1 and BA.4/5) was strong and stable over time, and no major differences were observed in ADCC activity against Omicron variants compared to the D614G Spike. Our work extends previous observations that showed strong ADCC activity after a fourth dose of mRNA vaccine against several Omicron variants, including BA.1, BA.2, BA.2.12.1, and BA.4/5, and that the ADCC responses were more stable than neutralization over time [38]. This might explain partially why vaccines remain effective, as the Fc-effector functions would have a significant role in vaccine efficacy. 

Finally, we noted that donors with recent BTIs elicited antibodies with better functional activities, not attributable to an enhancement in antibody level. However, we also observed that differences between the two groups were more pronounced for neutralization than for ADCC activity, thus indicating that vaccines could be improved to elicit more potent neutralizing antibodies against current variants. However, due to the small number of donors included in our study, these observations need to be confirmed with a larger number of individuals. 

Due to the decreased number of hospitalizations and deaths related to COVID-19, the World Health Organization declared in early May 2023 that SARS-CoV-2 is “now an established and ongoing health issue which no longer constitutes a public health emergency of international concern” [48]. However, our data suggest that health authorities must continue to monitor immune responses to these new variants to determine the level of immunity in the population and, if necessary, start vaccination campaigns tailored to the circulating variants, particularly to protect vulnerable populations.

## 5. Conclusions

Our results show that, even after five doses of the SARS-CoV-2 mRNA vaccine, neutralizing responses remain low against currently circulating variants. In contrast, ADCC responses are efficient against different Omicron subvariants. Our results also show that both neutralization and ADCC functions are largely improved by hybrid immunity.

## Figures and Tables

**Table 1:** Mutations and deletions in the Spike amino acid sequence from D614G and several Omicron subvariants compared to the ancestral strain.

Variants	Mutations in the Spike
D614G	D614G
BA.1	A67V, Δ69–70, T95I, G142D/Δ143–145, Δ211/L212I, ins214EPE, G339D, S371L, S373P, S375F, K417N, N440K, G446S, S477N, T478K, E484A, Q493R, G496S, Q498R, N501Y, Y505H, T547K, D614G, H655Y, N679K, P681H, N764K, D796Y, N856K, Q954H, N969K, L981F
BA.4/5	T19I, LPPA24–27S, Δ69–70, G142D, V213G, G339D, S371F, S373P, S375F, T376A, D405N, R408S, K417N, N440K, L452R, S477N, T478K, E484A, F486V, Q498R, N501Y, Y505H, D614G, H655Y, N679K, P681H, N764K, D796Y, Q954H, N969K
BQ.1.1	T19I, LPPA24–27S, Δ69–70, G142D, V213G, G339D, R346T, S371F, S373P, S375F, T376A, D405N, R408S, K417N, N440K, K444T, L452R, N460K, S477N, T478K, E484A, F486V, Q498R, N501Y, Y505H, D614G, H655Y, N679K, P681H, N764K, D796Y, Q954H, N969K
XBB	T19I, LPPA24–27S, V83A, G142D, Δ144, H146Q, Q183E, V213E, G339H, R346T, L368I, S371F, S373P, S375F, T376A, D405N, R408S, K417N, N440K, V445P, G446S, N460K, S477N, T478K, E484A, F486S, F490S, Q498R, N501Y, Y505H, D614G, H655Y, N679K, P681H, N764K, D796Y, Q954H, N969K
XBB.1.5	T19I, LPPA24–27S, V83A, G142D, Δ144, H146Q, Q183E, V213E, G252V, G339H, R346T, L368I, S371F, S373P, S375F, T376A, D405N, R408S, K417N, N440K, V445P, G446S, N460K, S477N, T478K, E484A, F486P, F490S, Q498R, N501Y, Y505H, D614G, H655Y, N679K, P681H, N764K, D796Y, Q954H, N969K
CH.1.1	T19I, LPPA24–27S, G142D, K147E, W152R, F157L, I210V, V213G, G257S, G339H, R346T, S371F, S373P, S375F, T376A, D405N, R408S, K417N, N440K, K444T, G446S, L452R, N460K, S477N, T478K, E484A, F486S, Q498R, N501Y, Y505H, D614G, H655Y, N679K, P681H, N764K, D796Y, Q954H, N969K
FD.1.1	T19I, LPPA24–27S, V83A, G142D, Δ144, H146Q, Q183E, V213E, G252V, G339H, R346T, L368I, S371F, S373P, S375F, T376A, D405N, R408S, K417N, N440K, V445P, G446S, F456L, N460K, S477N, T478K, E484A, F486P, F490S, Q498R, N501Y, Y505H, D614G, H655Y, N679K, P681H, N764K, D796Y, Q954H, N969K

**Table 2:** Characteristics of the SARS-CoV-2-vaccinated cohort.

		Vaccinated Cohort
**Number (n) ^a^**		18
Age ^b^		59 (51–64)
Sex ^a^	Female (n)	11
	Male (n)	7
Days between the fourth and fifth doses ^b^		148 (129–156)
Fifth dose (n) ^a^	Pfizer monovalent	2
	Moderna monovalent	0
	Pfizer BA.4/5	13
	Moderna BA.1	3
Days between the fourth dose and W3-Va4 ^b^		29 (23–40)
Days between the fourth dose and M4-Va4 ^b^		124 (116–133)
Days between the fifth dose and W3-Va5 ^b^		22 (21–27)

^a^ Values displayed are numbers. ^b^ Values displayed are medians, with interquartile ranges in parentheses.

**Table 3:** Characteristics of the donors with recent or no recent breakthrough infection.

		No Recent BTI	Recent BTI
**Number (n) ^a^**		9	9
Age ^b^		59 (50–67)	59 (49–63)
Sex ^a^	Female (n)	7	4
	Male (n)	2	5
Days between the fourth and fifth doses ^b^		151 (118–154)	147 (136–192)
Fifth dose (n) ^a^	Pfizer monovalent	1	1
	Moderna monovalent	0	0
	Pfizer BA.4/5	6	7
	Moderna BA.1	2	1
Days between the fourth dose and W3-Va4 ^b^		36 (24–43)	27 (21–38)
Days between the fourth dose and M4-Va4 ^b^		128 (118–148)	124 (111–129)
Days between the fifth dose and W3-Va5 ^b^		21 (21–25)	26 (19–37)

^a^ Values displayed are numbers. ^b^ Values displayed are medians, with interquartile ranges in parentheses. Continuous variables between individuals with no recent and recent BTIs were compared by using Mann–Whitney or unpaired t tests. A *p* < 0.05 was considered statistically significant for all analyses. No statistical differences between the two groups were found for any of the tested parameters.

## Data Availability

Further information, data reported in this paper, and requests for resources and reagents should be directed to and will be fulfilled by the lead contact, Andrés Finzi (andres.finzi@umontreal.ca), upon request.

## Supplementary Materials

The following supporting information can be downloaded at: https://www.mdpi.com/article/10.3390/v15091926/s1, Figure S1: Correlations between plasma binding and neutralization; Figure S2: Correlations between plasma binding and ADCC.
