# Supplementary material for: Humoral Responses Elicited after a Fifth Dose of SARS-CoV-2 mRNA Bivalent Vaccine
Source: Viruses. 2023 Sep 15;15(9):1926. doi: 10.3390/v15091926 (PMC10535273; doi:10.3390/v15091926)
Supplement: Supplementary file 1 [file viruses-15-01926-s001.zip › viruses-2577178-supplementary.pdf]

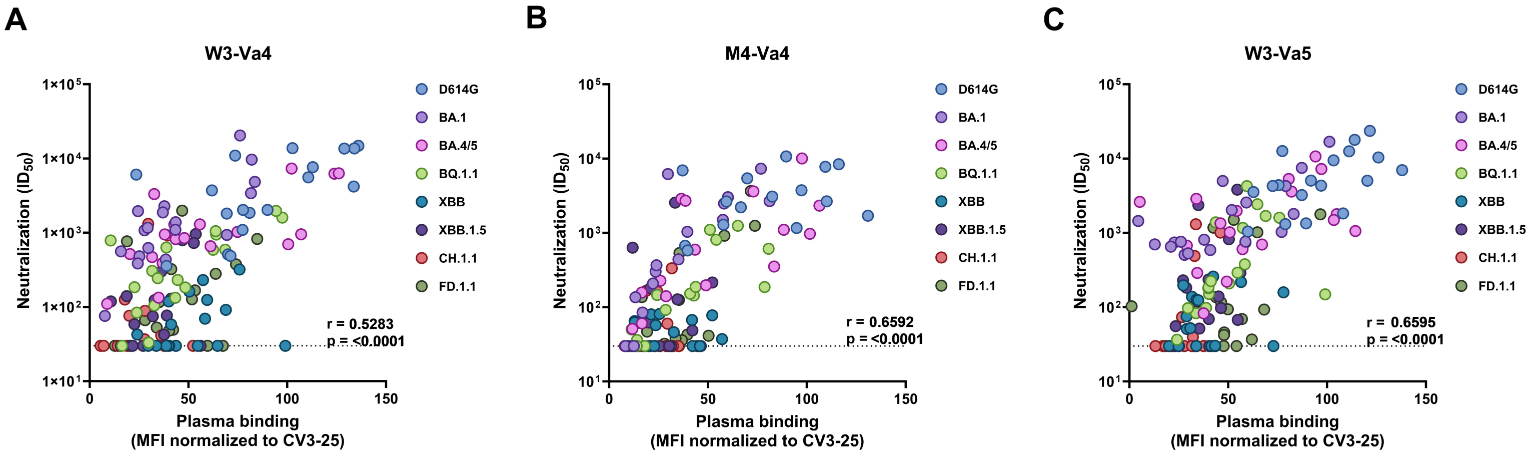

Figure S1. Correlations between plasma binding and neutralization. (**A**–**C**) The correlations between the capacity of plasma from vaccinated individuals collected at W3-Va4 (**A**), M4-Va4 (**B**) and W3-Va5 (**C**) to recognize Spike and the neutralization activity was calculated by nonparametric Spearman correlation.

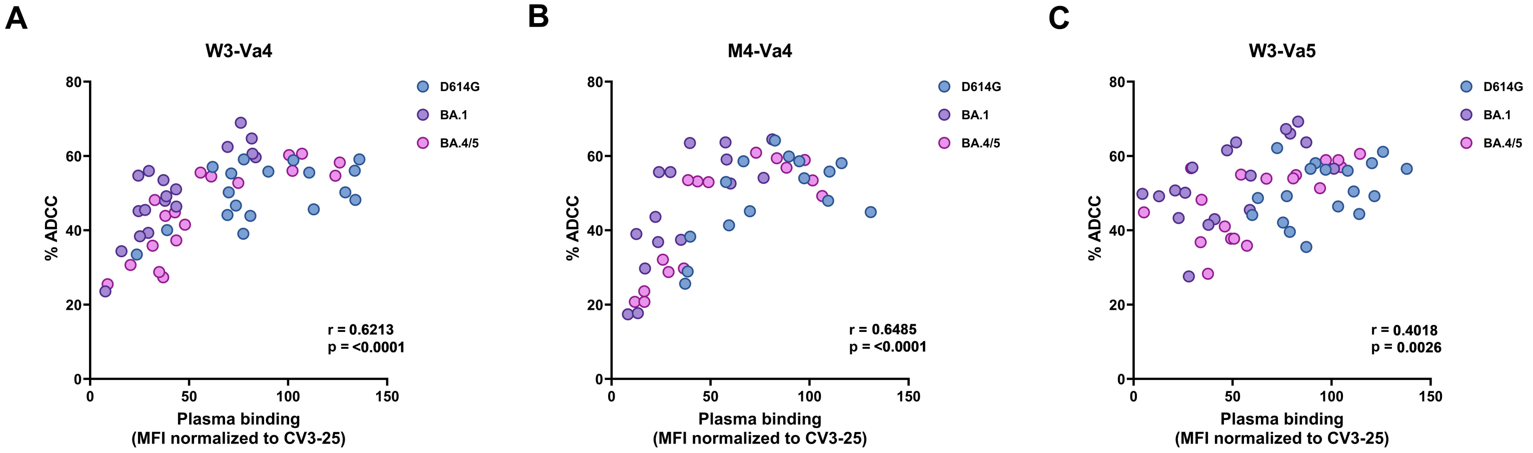

Figure S2. Correlations between plasma binding and ADCC. (**A**–**C**) The correlations between the capacity of plasma from vaccinated individuals collected at W3-Va4 (**A**), M4-Va4 (**B**) and W3-Va5 (**C**) to recognize Spike and the ADCC activity was calculated by Pearson correlation or nonparametric Spearman correlation.
